# An Evidence Theoretic Approach for Traffic Signal Intrusion Detection

**DOI:** 10.3390/s23104646

**Published:** 2023-05-10

**Authors:** Abdullahi Chowdhury, Gour Karmakar, Joarder Kamruzzaman, Rajkumar Das, S. H. Shah Newaz

**Affiliations:** 1 School of Computer and Mathematical Sciences, The University of Adelaide, Adelaide, SA 5005, Australia; 2 Centre for Smart Analytics, Federation University Australia, Ballarat, VIC 3350, Australia; 3 Institute of Innovation, Science and Sustainability, Federation University Australia, Ballarat, VIC 3350, Australia; 4 Information Technology Services, Federation University Australia, Mount Helen Campus, Ballarat, VIC 3350, Australia; 5 School of Computing and Informatics, Universiti Teknologi Brunei, Jalan Tungku Link, Gadong BE 1410, Brunei; 6 KAIST Institute for Information Technology Convergence, 291 Daehak-ro, Yuseong-gu, Daejeon 34141, Republic of Korea

**Keywords:** traffic signals, intrusion detection, intelligent transportation systems

## Abstract

The increasing attacks on traffic signals worldwide indicate the importance of intrusion detection. The existing traffic signal Intrusion Detection Systems (IDSs) that rely on inputs from connected vehicles and image analysis techniques can only detect intrusions created by spoofed vehicles. However, these approaches fail to detect intrusion from attacks on in-road sensors, traffic controllers, and signals. In this paper, we proposed an IDS based on detecting anomalies associated with flow rate, phase time, and vehicle speed, which is a significant extension of our previous work using additional traffic parameters and statistical tools. We theoretically modelled our system using the Dempster–Shafer decision theory, considering the instantaneous observations of traffic parameters and their relevant historical normal traffic data. We also used Shannon’s entropy to determine the uncertainty associated with the observations. To validate our work, we developed a simulation model based on the traffic simulator called SUMO using many real scenarios and the data recorded by the Victorian Transportation Authority, Australia. The scenarios for abnormal traffic conditions were generated considering attacks such as jamming, Sybil, and false data injection attacks. The results show that the overall detection accuracy of our proposed system is 79.3% with fewer false alarms.

## 1. Introduction

Traffic signals control traffic flows in different directions. This control reduces traffic jams and delays and bypasses incidents. Therefore, traffic signals are the heart of the city’s traffic management of an intelligent transportation system. Intelligent transportation systems (ITSs) aim at making reliable, efficient, and protected transportation systems by integrating smart transportation management and control systems through communication networks and computing techniques [1]. Reliable and efficient traffic signals save lives by bypassing incidents and responding to emergency vehicles so that they can meet the target response time [2,3]. Traffic controllers control the traffic signals dynamically based on the traffic conditions derived from wirelessly collected sensor data. These sensors are installed in external places, such as being buried in the roads and equipped with roadside units. These sensors, their communication systems, and traffic signals are vulnerable to cyber attacks. Hackers or cybercriminals can easily create traffic disruptions or diversions by compromising the traffic signals to achieve the benefits they are interested in [4,5]. The chance of hacking or intruding traffic signals is sharply increasing over time because the signals are being equipped with more and more smart electronic devices and are increasingly dependent on wireless and internet-based data communications. The cyber security threats in the traffic signals will culminate at the peak when Autonomous Vehicles (AVs) hit the city’s roads. AVs are a key component of an ITS. These AVs are equipped with advanced sensors and communication systems that allow them to operate without human intervention. Although there are many benefits of AVs, they are also vulnerable to cyber attacks because of their reliance on a range of connected devices and systems, such as GPS, sensors, and wireless communication networks. Hackers could potentially take control of an AV, causing it to malfunction or even crash [6,7,8].

The authors of [9] showed that disabling as few as seven signals in peak time periods for a couple of hours using a max vehicle affected model can cost around US USD 0.93 million, while the maximum vehicle flow targeting method can cost around US USD 0.98 million if 26 signals are impacted. The authors employed dynamic traffic assignment to show the consequences of traffic signal assaults and to mimic network-wide repercussions of intersection failures. The green traffic signal phase time analysis was also performed in [10]. It is imparted that the analysis of the patterns of green traffic signal phase time can be exploited to detect potential intrusion. However, in this study, a theoretical model for intrusion detection and its evaluation and validation were not specifically performed. In the current literature, there exist congestion-based attack analysis [11] and intrusion detection techniques [12] that exploit traffic image features. Additionally, with intrusion detection considering a specific attack, the other loopholes of these approaches rely on a selected set of traffic image features and an image prepossessing technique (background separation) that usually leads to being more computationally expensive. To advance the research in this area and introduce a more generic approach capable of detecting intrusion under various attacks, we introduced an approach for identifying traffic signal intrusion, which was published in [13]. However, the main problems of this approach are that important traffic signal parameter vehicle speed was not considered and mass value functions for flow rate and speed were not properly formulated. Additionally, there is uncertainty associated with probabilistic measures and converting the flow rate and vehicle speed data into normal distributions was not performed. By addressing these research issues, in this paper, we introduced a traffic signal Intrusion Detection System (IDS) with the following contributions:

- We developed a theoretical model for our proposed IDS, leveraging the Dempster–Shafer (DS) decision theory. Our model incorporates real-time observations of vehicle flow rate, vehicle speed, and traffic signal change duration, as well as relevant historical data obtained from transportation authorities. By utilising this decision theory, we sought to enhance the performance of our proposed system, leading to a more efficient and effective traffic control system. Our approach enables the integration of both historical and real-time data, facilitating the system’s ability to make more precise and informed decisions.
- The historical data associated with traffic flow and vehicle speed were transformed into normal distributions. A heuristic mass function was introduced by assigning higher probabilities to those phase time values that are most frequently observed and are closer to the most observed value.
- To assess the uncertainty associated with our observations, we utilised Shannon’s entropy. In order to verify and validate our proposed IDS, we developed a simulation model that is based on the SUMO traffic simulator [14]. Our simulation model incorporates real-world scenarios and utilises data collected by the Victorian Transportation Authority, Australia (VicRoads). The results of our simulations demonstrate that our proposed IDS is highly effective in detecting intrusions on traffic signals while reducing false alarms.

Since our proposed IDS is based on anomaly detection, it has the ability to detect intrusions created by the spoofed vehicles proposed in [11], as our method picks up any anomaly caused by intrusions, including intrusions in roadside sensors and traffic controllers and signals. Flow rate, phase time, and vehicle speed are affected by the attacks in a traffic signal irrespective of their types. Therefore, in contrast to image analysis techniques, as mentioned before, our IDS is more generic because it detects by identifying the inconsistencies between the traffic flows, vehicle speeds, and phase time of the observed and normal traffic conditions. In summary, the overall advantages of our proposed solution are: (i) it is more generic and comprehensive in response to the intrusion of various forms, and (ii) it does not require any image capture and analysis as required by the above studies.

The organisation of the paper is as follows. Section 2 presents the related works on the impact of attacks on traffic signals and intrusion detection in traffic signals. Our proposed IDS along with its theoretical modelling is provided in Section 3. Finally, the experimental results and conclusions are given in Section 4 and Section 5, respectively.

## 2. Related Works

To reduce traffic congestion and travel time, enhance traffic flow management, and protect the safety of road users, intelligent traffic systems, including traffic signals, are utilised [15]. Malicious disruption or manipulation of the lights of a traffic signal can result in catastrophic events such as massive delays, financial loss, and loss of life. Many traffic lights have recently been revealed to have been tampered with throughout the world, emphasising the necessity of precise intrusion detection. Since VANETs are important part of an ITS, a survey on machine learning based IDSs is presented in [16]. As VANET is an ad hoc vehicular network comprising vehicles and road side unites (RSUs), this survey did not consider IDSs for traffic signals.

Feng et al. [6] indicated that intruders could send malicious, spoofed, and corrupted data to the traffic signal control units. These exposures exhibit the pressing need to develop an intrusion detection system for traffic signals. So far, some research studies exist for assessing the impact of attacks on and detecting intrusions in traffic signals. These studies are presented in the following sections.

### 2.1. Intrusion in Traffic Signals

Intrusion attacks in intelligent traffic signals can cause significant safety hazards and traffic disruptions. Intrusion in traffic signals and traffic control systems refers to any unauthorised access, attack, or disruption of the system’s functionality, which can compromise the safety, privacy, and security of the system and its users. Intelligent transportation systems use advanced technology and communication networks to optimise traffic flow, reduce congestion, enhance safety, and improve overall transportation efficiency. These systems rely heavily on connected devices, sensors, data processing, and communication networks, making them vulnerable to cyber threats.

Intrusion threats in intelligent traffic signals can be launched by intruders from outside the network using various methods. Some common intrusion attacks include replay attacks, jamming attacks, Sybil attacks, and false data injection attacks. A jamming attack is an intrusion attack in which an attacker sends messages to a specific RSU, disrupting the communication between the targeted RSU and other RSUs in the network. This can prevent the RSU from receiving essential messages or updates, leading to potential safety hazards or traffic disruptions. Replay attacks intercept the messages exchanged between sensors in the ITS network and re-transmit them to impersonate and steal their identity. This can allow an attacker to gain unauthorised access to the network or impersonate a legitimate user, potentially leading to data theft or other malicious activities. Sybil attacks are another type of intrusion attack where an attacker uses multiple identities to deceive other sensors by reporting fake road congestion. By flooding the network with false information, the attacker can disrupt traffic flow or cause congestion in specific areas, leading to potential safety hazards or traffic disruptions. False data injection is another type of intrusion attack, where an attacker sends false information about the current traffic situation on the road. This can lead to traffic disruptions or congestion, potentially causing safety hazards or delays for drivers.

A ransomware attack hit San Francisco’s Municipal Transportation Agency in November 2016, preventing the agency from collecting fares and leading to several key systems shutting down. The attackers demanded a ransom payment of 100 Bitcoin (around USD 73,000 at the time) to restore the systems [17].

The city of St. Louis’ traffic system was hacked, causing the city’s traffic lights to go dark for several hours. The attack disrupted traffic and caused several accidents, highlighting the potential danger of hacking into critical ITS infrastructure [18].

Implementing robust security measures to prevent unauthorised access and manipulation is important, including access controls, encryption, network segmentation, and ongoing monitoring and testing of their systems to identify and address vulnerabilities.

### 2.2. Evaluating the Impact of an Attack on Traffic Signals

The U.S. Department of Transportation (USDoT) uses the Intelligent Traffic Signal System (I-SIG) to increase traffic management efficiency, reduce traffic congestion, and increase safety [19]. An approach presented in [20] to perform a data spoofing attack on I-SIG demonstrated that the data spoofing could be highly effective in manipulating the signal control algorithm. The spoofed trajectory analysis of the study shows that one single attack could increase the traffic delay by up to 68.1%. Introducing I-SIG improves traffic mobility by 26.6%, but only a single attack can make the traffic condition 23.4% worse than the traffic condition without using I-SIG [21]. Anomalies in traffic control systems not only increase traffic congestion but can also contribute to substantial financial loss. An intruder was able to attack the security cameras used in the Carmel Tunnels toll on a major road network in Haifa, Israel, on 8 September 2013. This cyber attack caused around eight hours of disruption, created enormous logistic issues, and cost hundreds of thousands of dollars [22].

### 2.3. Detecting Intrusions in Traffic Signal

Traffic congestion is considered a severe challenge to urban areas as it harms economic growth, increases gas emissions, and may lead to increased numbers of accidents [23]. Therefore, intelligent traffic signal management is crucial. The algorithms governing intelligent traffic signals are vulnerable to connected vehicle data spoofing attacks, resulting in the creation of congestion at road intersections. To overcome this problem, the foremost important step would be timely and accurate congestion attack detection and identification.

Xiang et al. [11] applied empirical studies to detecting intrusions in traffic signals. From a range of traffic images (vulnerable to potential data spoofing attacks), the solution measures the traffic flow characteristics and predicts whether there would be any attacks that would be able to cause congestion with a certain probability. Additionally, to verify any congestion occurrence, the authors proposed a tree-regularised gated recurrent unit-based approach that analyses the underlying relation between the congestion attack and traffic flow features at a given time. Therefore, when there is an attack that may lead to congestion with high probability, as well as if any congestion development is observed from the subsequent traffic flows, a congestion attack can be inferred. Based on an experiment, the authors imparted that the solution performs well in terms of attack detection accuracy and timeliness. They also proposed to validate their prediction by using traffic parameters in an intersection to determine if congestion really occurs as a result of placing spoofed vehicles in the intersection. Although this model can detect intrusion created by spoofed vehicles, it cannot detect intrusion created by malicious users modifying the data through unauthorised access to the traffic controller unit. This scenario will create congestion in intersections without having any indication from the image generation model.

The authors of [24] highlighted that the existing solutions for identifying congestion attacks have the disadvantage of delay. Considering that a congestion attack is made continuously and periodically, the authors developed a solution for predicting congestion attacks and attack frequency and the timely forecast of future congestion attacks. The prediction mechanism uses supervised machine learning algorithms where historical data are fed in. The solution collects traffic flows of variable spoofing frequencies and, then, the flows that lead to congestion are used to extract important features. It implements ensemble learning to establish correlations between various traffic flow features and abnormal congestion and attack frequency. The authors demonstrated the significance of their solution based on experimental results.

Li et al. [12] introduced a traffic image feature-based attack prediction mechanism. They claimed that, based on feature-based learning, it is possible to understand the relationship between attack and the caused congestion. Using a cycle generative adversarial network, the authors proposed a prediction mechanism where L1 regularisation loss is made to understand and recognise the difference between the last-vehicle and the first-vehicle attack.

Even though the image analysis technique was adopted as a universal technique for road safety, the 5th-generation adaptive traffic signal management system [25] employs self-learning capabilities and real-time computations to enhance the quality of traffic management for both conventional and autonomous vehicles by observing real-time traffic situations using different in-road sensors and RSUs. The traffic management system can independently learn traffic management strategies and make wise choices to optimise traffic flow, reducing the computational decision-making load incurred by image analysis techniques.

Most Adaptive Traffic Systems (ATSs), such as SCATS [26], SCOOT [27], and OPAC [27], use sensors to count vehicles and pedestrians and monitor vehicle speed. These ATSs use vehicle count sensor data, average vehicle speed data, and cell arrival and departure data. Most traffic signal intrusions occur by compromising roadside sensors [28]. So, it is crucial to develop a traffic signals intrusion detection system that utilises data collected from roadside sensors rather than relying on image analysis techniques. Other inherent issues associated with image analysis techniques are selecting optimised feature sets and separating background images, further contributing to its high computational requirements.

The framework developed by the authors of [10] allows for the identification of potential attacks on traffic control systems through the use of visual analytics and data collected from traffic signals. This framework provides a comprehensive overview of traffic signal data by breaking down traffic light cycles and calculating statistics on their duration and distribution over a given time period. By normalising individual traffic light cycles, the framework can detect potential abnormal patterns, making it possible to differentiate normal from abnormal traffic signal behaviour. This method of analysis can be an effective tool for identifying and mitigating potential security threats to traffic control systems.

The paper [13] describes our initial work on detecting intrusions in traffic signals by analysing vehicle flow rate and phase time at intersections. The DS decision theory was used to combine the evidential observations of these two traffic characteristics, using historical data provided by the Victorian Transportation Authority in Australia (VicRoads) and the SUMO traffic simulator to simulate intruded and typical traffic scenarios. We evaluated the performance of the proposed IDS under both normal traffic conditions and induced intrusions.

However, the intrusion detection approach described in [13] has some limitations. Firstly, it does not consider vehicle speed, which is an important traffic characteristic. Moreover, the mass value function defined for flow rate and phase time is not adequately specified, since the minimum value of the mass function is set to 0.5, whereas in reality, the mass function values should range from 0 to 1. Additionally, it does not address the uncertainty associated with the signals being normal or abnormal. This paper focused specifically on addressing these practical research issues to improve the accuracy and effectiveness of intrusion detection in traffic signals.

## 3. Proposed Intrusion Detection System

Anomaly-based intrusion detection can be particularly effective for detecting new or unknown threats that may not be detected by signature-based intrusion detection methods, which rely on known patterns or signatures of known threats. Anomaly-based detection is useful because it can detect previously unknown attacks and it does not require prior knowledge of the attack signature. For detecting an intrusion in an intelligent traffic signal, these merits motivated us to propose an anomaly-based intrusion detection system in this paper. This system involves creating a baseline of normal traffic patterns and behaviour using historical traffic data and then monitoring the traffic for any deviations from this baseline that indicates a potential intrusion.

The implementation of anomaly-based intrusion detection for intelligent traffic systems involves several steps:

- Data collection: Traffic data are collected from the intelligent traffic system, including information on traffic flow rate, vehicle speed, and phase time.
- Baseline generation: A baseline is generated from the collected traffic data, which represents the normal traffic patterns and behaviour for the system.
- Anomaly detection: The traffic data are continuously monitored for any deviations from the baseline. Significant deviation from the baseline is flagged as potential anomalies and investigated further.
- Response: Once an anomaly is detected, the system sends an alarm to the traffic controller.

Details of our proposed model are given below.

### 3.1. Overview of the Proposed IDS

**Figure 1:**
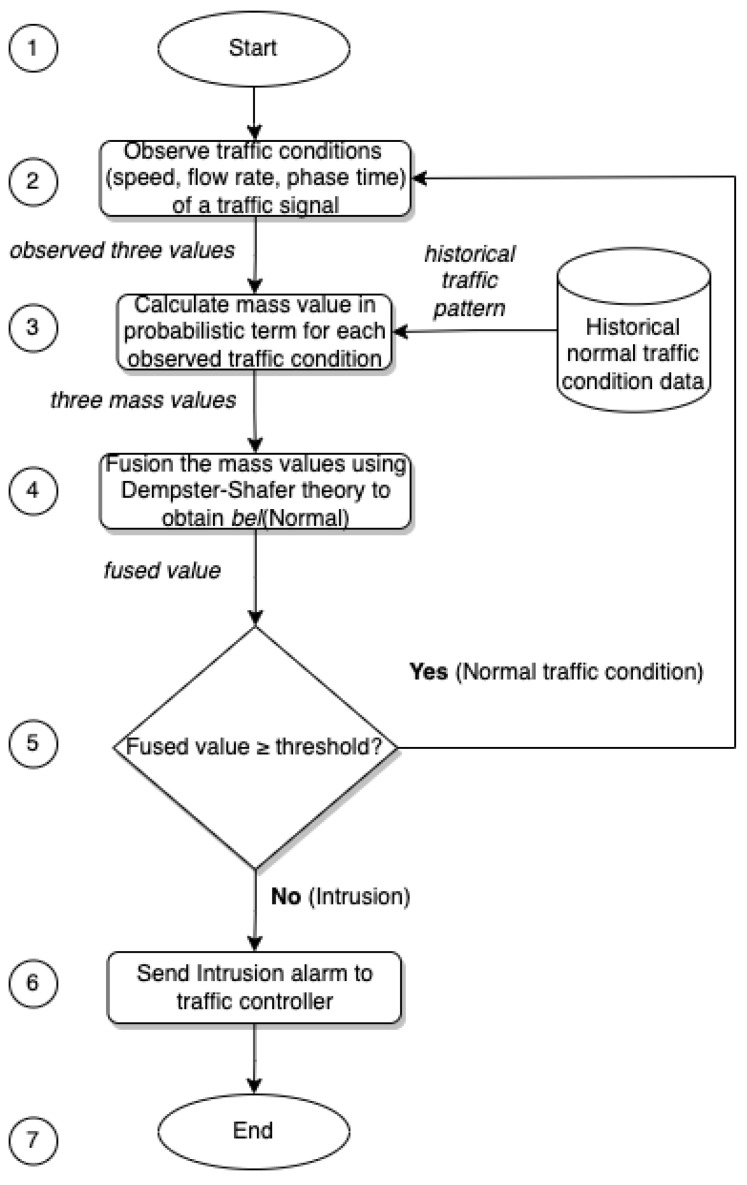
The operational flow chart of the proposed IDS.

Our proposed system primarily monitors the state of a current traffic light, which is statistically calculated based on the flow rate, vehicle speed, and the traffic signal’s phase time. The current state of the traffic signal is compared and contrasted with the relevant status of the traffic signals obtained from the related historical data collected by its Traffic Management System (TMS) [29] infrastructure to determine if it is functioning normally or abnormally. The operational flow chart of the proposed IDS is depicted in Figure 1. There are seven steps in our proposed intrusion detection system. The intrusion detection process starts in Step 1. In Step 2, our system collects data on road traffic, including flow rate, average vehicle speeds, and phase time. The details of the simulation used for the data collection are given in Section 4.1. In the third step, the mass values in probabilistic terms for each traffic condition (flow rate, vehicle speed, and phase time) are calculated. Section 3.3, Section 3.4 and Section 3.5 describe the details of this step. The mass values are then fused to find the belief value of the normal traffic condition in the fourth step. In the next step, the system checks if the fused value equals or exceeds the threshold value (defined in Section 4). If the value is above the threshold value, the traffic condition is determined to be normal and the system continues observing the traffic data. If the value is below the threshold, our system goes to Step 6 and sends an intrusion detection alarm to the traffic controller. Step 7 ends the intrusion detection process.

### 3.2. Monitoring the Status of the Traffic Signal

Our suggested technique starts the intrusion detection assessment process in the second step by using a historical traffic pattern probability mass function that has not been tampered with by an intruder at a certain moment inside a specific time window (e.g., from 08:00 a.m. to 09:00 a.m. on Monday).

We acquired all relevant data from VicRoad’s traffic data [30] for this research because we wanted to use previous data to monitor the state of a current traffic light at a certain moment. After this, we go through how to estimate the probability mass function of these data for a certain time window.

In the second phase, we need to calculate the continuous observed values of the traffic signal attributes for intrusion detection. We selected the three most important traffic attributes, namely traffic flow rate, signal phase time, and average vehicle speed. Attackers can change the phase time information manually and/or change the phase time by changing the flow rate information in an intersection and/or modify the average vehicle speed to falsify the traffic data. For example, hackers can extend the signal phase time to create disruption for plotting terrorist activity or a thief can extend a green signal phase time to pass quickly by a stolen vehicle. Vehicle speed was selected as it is also profoundly affected by traffic signal disruption. The impact of their changes only stays for one or two cycles, not being sufficient to create considerable disruption.

To determine whether a traffic signal is functioning normally or abnormally, we employed an inference method based on DS decision theory [31]. This theory, which is an extension of Bayesian theory, provides distributed support for various propositions based on temporal evidence. Our system defines the frame of discernment using three propositions: *N*, ¬N, and (N∨¬N), which represent the current observation being normal, not normal, and uncertain, respectively.

Since flow rate, vehicle speed, and phase time are measured by individual sensors, the belief function contributing to each proposition must be statistically measured for each sensor. Let $R_{jw}$ denote the id of a sensor located at intersection *j* with sensor type *w*, where w∈F,S,P represents the observed events of flow rate, vehicle speed, and phase time, respectively. As these events are observed over a time period for a given intersection, we utilised probability mass functions based on historical data from the corresponding time window of that day to determine the probability of a particular observation is normal. These time windows may differ for working and non-working days and are typically one hour in duration (e.g., 08:00 a.m. to 09:00 a.m. or 09:00 a.m. to 10:00 a.m.). The lower limit of the probabilistic value of $j^{\mathrm{th}}$ intersection being normal for *y* events can be defined using the belief function of the DS theory [32]:

(1) $$bel_{j}\left(N\right)=\frac{1}{1-k}\times \sum_{\cap E_{w}\left(t\right)=N\neq \emptyset }\;\prod_{1\leq w\leq y}m_{j}\left(E_{w}\left(t\right)\right),$$

where *k* is defined as:

(2) $$k=\sum_{\cap E_{w}\left(t\right)=\emptyset }\;\prod_{1\leq w\leq y}m_{j}\left(E_{w}\left(t\right)\right).$$

According to the Shannon information theory [33], the uncertainty is the highest when $m_{j}\left(E_{w}\left(t\right)\right)=m_{jw}\left(N\right)$ = 0.5. Note, here, $m_{jw}\left(N\right)$ denotes the probabilistic value of a mass function for $w^{\mathrm{th}}$ event ($E_{w}\left(t\right)$) with the $j^{\mathrm{th}}$ intersection being normal. If the value of $m_{jw}\left(N\right)$ moves in either direction from 0.5, the uncertainty decreases. Applying the principle of Shannon information theory, the uncertainty associated with $m_{jw}\left(N\right)$ i.e., the probability, $m_{jw}$(N∨¬N) is defined as:

(3) $$\begin{array}{c} m_{jw}\left(N\vee \neg N\right)=-m_{jw}\left(N\right)log_{2}m_{jw}\left(N\right)-\\ \left(1-m_{jw}\left(N\right)\right)log_{2}\left(1-m_{jw}\left(N\right)\right). \end{array}$$

Since $m_{jw}\left(N\wedge \neg N\right)$ denotes the null hypothesis, i.e., $m_{jw}\left(N\wedge \neg N\right)$ = 0, $m_{jw}\left(\neg N\right)$ is derived as,

(4) $$\begin{array}{c} m_{jw}\left(\neg N\right)=1-m_{jw}\left(N\right)-m_{jw}\left(N\vee \neg N\right). \end{array}$$

The upper limit (plausibility) of the $j^{\mathrm{th}}$ intersection being normal is defined as:

(5) $$pl_{j}\left(N\right)=1-bel_{j}\left(\neg N\right).$$

For obtaining the uncertainty value and then the belief value using (3) and (1), respectively, we need to calculate $m_{j}\left(E_{w}\left(t\right)\right)$ for the flow rate, vehicle speed and phase time.

An attacker may attempt to intrude on one or more of the sensors used to collect data for the TMS. To detect whether a sensor has been compromised, we can compare its observed values with the corresponding original (i.e., unaltered) historical values. This process involves the development of probability mass functions, denoted by $m_{j}\left(\right)$, which are used in the DS theory-based fusion approach defined in (1) and (5). By comparing observed and historical values, our approach enables the identification of abnormal or anomalous sensor behaviour, which can be indicative of a security breach.

### 3.3. Development of the Probability Mass Function

We developed a formula (refer to (6)) to calculate the mass value that identifies an intrusion from a regular traffic signal based on the assumption that the underlying historical data are normally distributed. In the following section, we present an analysis of the validity of such assumptions and converted the data to follow normal distributions when the assumption did not hold true.

### 3.4. Data Conversion into Normal Distribution

**Figure 2:**
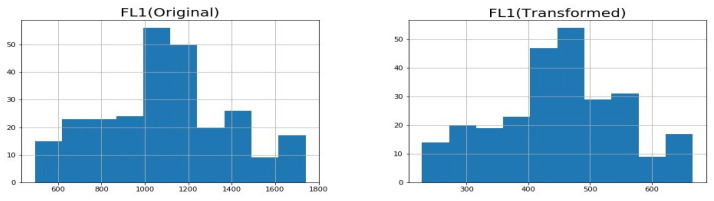
Histograms for flow rate for original and transformed data.

**Figure 3:**
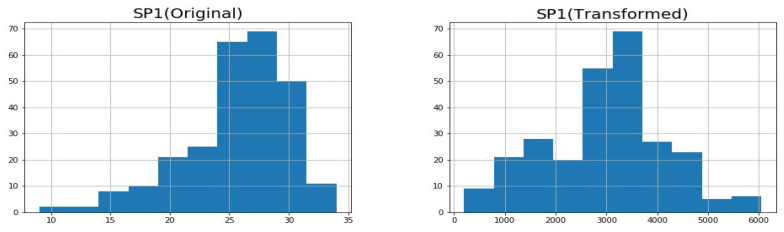
Histograms for vehicle speed original and transformed data.

As discussed in the previous section, we assumed that the traffic data follow the normal distribution. However, since the traffic data are not normally distributed, we transformed the data so that they follow the normal distribution. For example, the flow rate and vehicle speed distributions before and after normal transformation for Intersection 1 are shown in Figure 2 and Figure 3.

We applied the BoxCox transformation [34], which can transform the data to a normal distribution. The following equation defines BoxCox transformation.

(6) $$y=\left\{\begin{array}{cc} \frac{\left(x^{\lambda }-1\right)}{\lambda } & \mathrm{for}\;\lambda \neq 0\\ log(x) & \mathrm{for}\;\lambda =0. \end{array}\right.$$

Here, *x* and *y* represent data before and after the BoxCox transformation, respectively, and λ is a transformation parameter.

The calculation method for the probability of the observed evidence (e.g., flow rate, vehicle speed, and phase time) developed from the historical data using the probability mass functions is described below.

### 3.5. Probabilities of Observed Evidence for Flow Rate and Vehicle Speed

For an event *w* and the observed value $x_{w}$, the probability of the evidence can be calculated using (7).

(7) $$\begin{array}{c} m_{j}\left(x_{w}\right)=\left\{\begin{array}{c} 1-\frac{\sqrt{2}}{\sqrt{\pi }}\int_{0}^{z_{w}}e^{-\frac{x^{2}}{2}}dx\;\mathrm{if}\;z_{w}\geq 0\\ 1-\frac{\sqrt{2}}{\sqrt{\pi }}\int_{z_{w}}^{0}e^{-\frac{x^{2}}{2}}dx\;\mathrm{otherwise}, \end{array}\right. \end{array}$$

where $z_{w}=\left(x_{w}-\mu_{w}\right)$/$\sigma_{w}$, $x_{w}=E_{w}\left(t\right)$, and $\sigma_{w}$ and $\mu_{w}$ are the standard deviation and mean of the probability mass function, respectively.

### 3.6. Probability of Observed Evidence for Phase Time

The probability of observed phase times was computed. As the phase times observed take values from a limited set, they usually do not follow the normal distribution. Consequently, the probabilities were computed based on the frequency of observed phase times and the relative distance between an individual phase time with the most observed one. In calculating the probabilities, we used the two following rules, i.e., (i) assigning higher probabilities to those phase time values that are more frequently observed, and (ii) assigning higher values to those that are relatively closer to the most-observed value. The following equations capture such probabilities:

(8) $$\begin{array}{c} m_{j}\left(x_{w}\right)=\left\{\begin{array}{c} \alpha -\alpha \times \frac{\left(min\left(X\right)-x_{w}\right)}{min(X)}\;\mathrm{if}\;x_{w}<min\left(X\right)\\ \alpha \times \frac{\left(CT-x_{w}\right)}{CT}\;\;\;\;\mathrm{if}\;x_{w}>max\left(X\right)\\ e^{\left(\frac{A+B}{2}-1\right)}\;\;\;\;\;\mathrm{if}\;min\left(X\right)\leq x_{w}\leq max\left(X\right), \end{array}\right. \end{array}$$

where

(9) $$A=\frac{f(x_{w})}{max(f(X))}$$

and

(10) $$B=(1-\frac{\left|(x_{w}-argmax\left(f\left(X\right)\right))\right|}{max(X)-min(X)}).$$

Here, *A* and *B* are the two rules described above capturing the frequency of occurrence and the relative distance of an observation from the most frequently observed one. min(X) and max(X) represent the minimum and maximum phase time values observed in the historical data. $f(x_{w})$ and f(X), respectively, represent the frequency of the phase time value $x_{w}$ and the frequency distribution of all phase times observed in the historical data *X*. argmax(f(X)) represents the phase time that occurred most in the data set. Finally, CT represents the cycle time with a typical value of 180, and α is a chosen parameter that controls the behaviour of the probability function defined in (8), when the phase time observation falls beyond the minimum and maximum values in the historical data and α is chosen as 0.4.

## 4. Evaluating the Traffic Signal to Detect Intrusion

In the next step, we evaluated the status of a traffic signal to detect an intrusion in that traffic signal. The standard behaviour profile of a traffic signal was determined using the evaluated status of that traffic signal. Once the value of current events (e.g., $E_{F}\left(t\right)$, $E_{S}\left(t\right)$, $E_{P}\left(t\right)$) are determined, their probabilities can be calculated as $m_{j}\left(E_{F}\left(t\right)\right)$, $m_{j}\left(E_{P}\left(t\right)\right)$, and $m_{j}\left(E_{S}\left(t\right)\right)$ using (7) and the μ and σ of their corresponding probability mass functions. After this, the probability of being *N*, i.e., $bel_{j}\left(N\right)$ defined in (1), needs to be determined. If $bel_{j}\left(N\right)\geq \Phi$, the traffic signal of $j^{\mathrm{th}}$ intersection is considered to be normal, otherwise, it is not normal (intruded). Here, Φ is an intuitively-selected threshold that is used in Step 5 of our model (ref to Figure 1). The performance metrics of our proposed methods depend on the value of Φ. In an ideal case, the value of Φ can be considered 0.5.

### 4.1. Simulation Environment

We used the map of Melbourne CBD and VicRoads’ traffic data [35] for five critical intersections and created a simulation environment in SUMO using a popular microscopic traffic model presented in [14] and the Krauss car following model [36,37]. The selected intersections were (i) Lonsdale and Russel Street, (ii) Collins and Kings St, (iii) Elizabeth and Latrobe St, (iv) Collins, and Swanston St, and (v) Flinders and Swanston St Melbourne. For traffic data, we selected the peak-time traffic data from 08:00 a.m. to 10:00 a.m. Monday.

Normal and intrusion scenarios: for normal traffic conditions, the flow rate, vehicle speed, and phase time of an intersection for a particular scenario were derived through our above-mentioned simulation model developed using SUMO. The traffic distributions were initiated with the respective and normal (non-compromised) historical traffic information obtained from the VicRoads online data [30] in our simulation environment. To emulate the current traffic in the simulation, the density, vehicle speed, and phase time of the incoming and outgoing traffic of an intersection of interest were randomly selected from the range of the [minimum, maximum] value of the respective historical data for that day and time period collected from three years (2016–2018) of data available on the VicRoads website. Traffic volumes for the freeways and arterial roads data were taken from [38], signal details for simulation were taken from [39], and traffic signal volume data were taken from [40].

**Figure 4:**
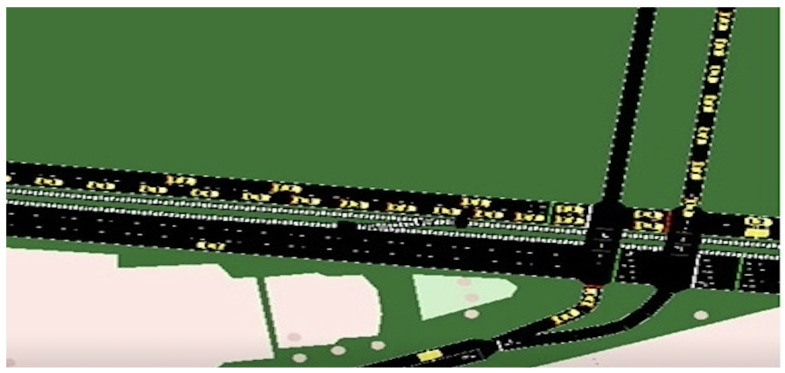
The traffic condition of the simulation environment for the normal scenario.

**Figure 5:**
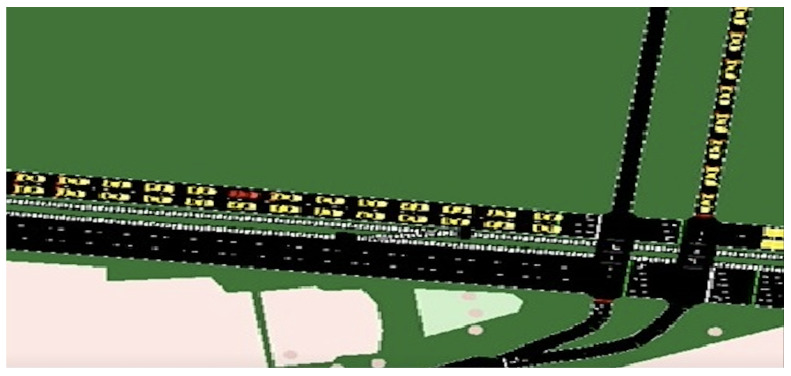
The traffic condition of the simulation environment for the intruded scenario.

Figure 4 and Figure 5 show the traffic flow of Intersection 1, namely Lonsdale and Russel Street, for its normal and intruded conditions, respectively.

As described in Section 2.1, traffic signals can be the victim of the intrusion. For simulating intrusions to the traffic signals, the flow rate of an intersection and/or average vehicle speed for a particular scenario was changed by intuitively induced phase time and vice versa. The induced average vehicle speed of an intersection was also inserted while phase time and the flow rate were normal. If the intersection is intruded for a very short time (e.g., less than one cycle time), flow rate, vehicle speed, and phase time data will remain within the range of 68% to 95% confidence intervals. If the duration of the intrusion is higher, flow rate, vehicle speed, and phase time will go outside the 95% confidence interval. To consider both cases (e.g., short and long time intrusion), the flow rate, vehicle speed, or phase time were induced in such a way that they remain within 68% to 95% confidence intervals in some cases (Scenario 2 and Scenario 4) and outside 95% confidence intervals of the relevant historical data for the other cases.

We used historical data to observe the current status of a traffic signal for a given point in time. Four different scenarios were created to test 40, 41, 39, 42, and 44 pieces of evidence for Intersection 1, Intersection 2, Intersection 3, Intersection 4, and Intersection 5, respectively. Scenarios 1 to 4 described in Section 4.3 show how different types of evidence (e.g., flow rate, phase time, and vehicle speed) were used. Using the normal historical data taken from the VicRoads website [30], initially, we simulated normal traffic scenarios. We simulated different malicious and abnormal traffic situations. The abnormal situations include incidents, vehicle breakdowns, unusual vehicle stops, and malicious driving behaviour to see the impact of these scenarios on traffic conditions. Note that, in normal traffic conditions, vehicle breakdown creates a traffic jam which reflects the jamming attack. Using Sybil attack, vehicles can be stopped or redirected. Therefore, consideration of unusual vehicle stops is regarded as the impact of a Sybil attack. By injecting false data in GPS or traffic signals, the driving behaviour of a vehicle can be manipulated, representing the false data injection attacks in the situations used in abnormal traffic scenario generation. Abnormal traffic scenarios (Scenario 2 and 4) were created considering the types of attacks mentioned here affecting flow rate, vehicle speed, and phase time of a traffic signal.

The simulation was modified in six different ways to create intruded scenarios. These modifications are described below:

Type 1: Congestion attack scenario

The number of vehicles on the route was falsely increased by modifying the following parameters. This modification would create an incorrect flow rate and cause the traffic controller to miscalculate the average speed. This scenario represents congestion attacks because it falsely increases on-road traffic.

Type 2: False vehicle number injection scenario

By changing the state of the lane area detector, the actual number of vehicles on the detector is overridden, leading to miscalculation of congestion on the cell and disrupting the dynamic adaptation of the traffic light phases.

Type 3: Replay attack scenario

Induction loop state values were replayed at certain intervals and transmitted to the regional traffic controller. This attack modifies flow rate count and average vehicle speed.

Type 4: Sybil attack scenario

The simulation environment is tampered by introducing a multitude of fake vehicles and sensors, which sent erroneous traffic data to the traffic controller system in such a manner that may create congestion on the roadways.

Type 5: False traffic light state injection

A false traffic light state was injected to modify the phase times, creating an impact of false traffic control.

Type 6: Jamming attack scenario

The lane attributes can be changed by transmitting false values or man-in-the-middle attacks. Examples of such attributes include the allowed speed limit, permitted vehicle categories, restricted vehicles, acceptable turns, and lane change permissions. These changes force vehicles to make incorrect turns, travel at varying speeds, and prevent certain vehicles from entering the lane.

The above scenarios consider most attacks that were previously observed in traffic signal intrusions (see Section 2.1).

### 4.2. Performance Metrics

Widely used standard performance metrics such as specificity, sensitivity, overall accuracy, and F-Score were employed to evaluate our model. If the normal (non-intruded) event is detected correctly, it is considered a True Positive (TP). Conversely, when an intruded event is detected as normal, it is marked as False Positive (FP). When the system is able to detect the intruded event correctly, it is considered a True Negative (TN), but when the system detected a normal event as an intruded event, it is considered a False negative (FN) value. The accuracy, sensitivity, specificity, and F-Score are defined as follows:

(11) $$Accuracy=\frac{TN+TP}{TP+TN+FP+FN};$$

(12) $$Sensitivity=\frac{TP}{TP+FN};$$

(13) $$Specificity=\frac{TN}{TN+FP};$$

(14) $$F-Score=2\times \frac{Sensitivity\times Precision}{Sensitivity+Precision}.$$

### 4.3. Results and Analysis

We used 206 observations for the five intersections for each piece of evidence (FP, FS, PS, and FPS) and scenario. Therefore, the total number of observations we used was 206×4×4=3296. These 206 observations were distributed to 40, 41, 39, 42, and 44 observations for Intersections 1 to 5, respectively. As a representative sample, Table 1, Table 2, Table 3 and Table 4 show the probabilities of signals being normal (*N*), not normal (¬N), and the uncertainty (N,¬N) for Scenarios 1–4 having various flow rates, average speeds, and phase times. Note, Scenarios 1 and 3 were created using the original data collected from VicRoads to emulate normal traffic conditions without intrusion. Scenarios 2 and 4 were created to induce intrusions by using the same data but manipulating a combination of flow rate, phase time, and average vehicle speed.

We used four different types of combinations for two different observations of an intersection. Four different combinations of two observations are (i) flow rate and phase time (FP), (ii) flow rate and vehicle speed (FS), (iii) phase time and vehicle speed (PS), and (iv) flow rate, phase time, and vehicle speed (FPS).

Table 1 and Table 3 present the intrusion detection results and their uncertainty values produced by our system for Scenarios 1 and 3, where no intrusions happened in any of the five intersections. Note, since uncertainty values are shown separately in all tables, throughout the chapter, belief values obtained by (1) denote the results. In contrast, Table 2 and Table 4 show the results for Scenarios 2 and 4 where intersections were intruded. Therefore, Scenarios 1 and 3 represent normal intersections, while Scenarios 2 and 4 are for the intruded intersections. The probability values highlighted with bold text represent which combination provides the best result. It also shows the combinations that have the lowest uncertainty.

#### 4.3.1. Detection Probability

In the case of intrusion in an intersection, the results produced by combining two pieces of evidence (e.g., FP, FS, PS) provide better detection than the results achieved using all three pieces of evidence (FPS). For example, in Table 4, our proposed method obtained the maximum detection probability values (e.g., 0.74, 0.72, 0.921, 0.73) for being not normal for four intersections using FS, while for Intersection 5, it (e.g., 0.62) was for FP. Table 4 also shows that the lowest uncertainty values (e.g., 0.06, 0.10, 0.07, 0.04) were obtained for the maximum number of intersections (4/5) using FS. Table 2 also supports a similar trend in the results. The detection probabilities obtained using FPS for Intersection 3 of Scenario 2 (see Table 2) and Intersection 5 of Scenario 4 (refer to Table 4) are 0.43 and 0.40, respectively, which are lower than the selected threshold 0.50, indicating the ineffectiveness of exploiting all three pieces of evidence for capturing intrusion. These results indicate that phase time is a major factor in intrusion detection. If a hacker can intrude on the sensor to change the phase time, it creates more congestion. Phase time impacts the traffic flow and average vehicle speed immediately. In the simulation, Intersection 5 and Intersection 2 had intruded phase time in Scenario 2 and Scenario 4, respectively. For Intersections 5 and 2, Table 2 and Table 4 present the high detection values exhibiting the efficacy of phase time consideration in spotting intrusion.

When the intersection is not intruded, i.e., normal, a combination of all three pieces of evidence (FPS) provides better detection results than the others. These results are above 0.50 for all intersections of the two scenarios. For example, for FS, the probability value for Intersection 3 shown in Table 1 is 0.48, while it is 0.58 for FPS. Table 3 shows that the probability values for Intersection 2 are 0.42, 0.48, and 0.50 for FP, FS, and PS, respectively, compared with 0.51 for FPS. These superior detection outcomes vindicate the use of FPS (three or more pieces of evidence) for the accurate detection of normal traffic conditions. The only loophole of FPS utilisation is that its uncertainty value is higher than that of others but not that high as its maximum value of 0.12. This higher uncertainty value is justified by the fact that, in a normal scenario, the real traffic conditions are dynamic. Traffic conditions may change due to many different reasons, such as different times (peak, off-peak), weather conditions, special events (e.g., road works, sports events), and school time. Flow rate, average vehicle speed, and phase time are affected because of the dynamic characteristics of the traffic conditions.

From the results discussed so far, it is evident that FPS is effective at identifying normal traffic conditions, but not so effective for intruded intersection detection. However, if we use the maximum probabilistic values, highlighted in bold, of an intersection in Table 1, Table 2, Table 3 and Table 4, our proposed system is able to detect the correct state (normal or intruded) of all intersections. For example, for intersections being normal, the maximum values for Intersection 3 in Table 1 and Intersection 2 in Table 3 are max (0.48, 0.52, 0.67, 0.58) = 0.67 and max (0.42, 0.48, 0.43, 0.51) = 0.51, respectively. Note, since for both intersections, these two maximum values (0.67 and 0.51) are above 0.50, our proposed system can detect normal traffic conditions accurately.

**Figure 6:**
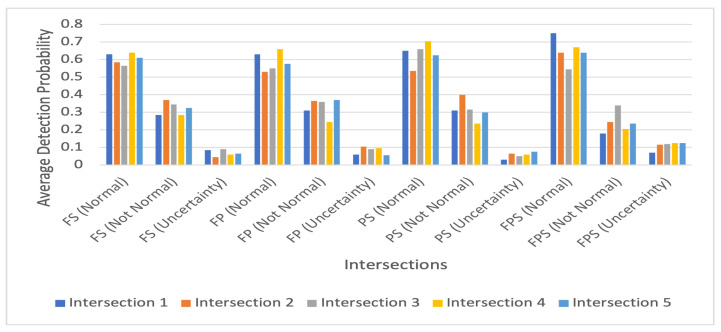
Average detection probability for normal intersections.

**Figure 7:**
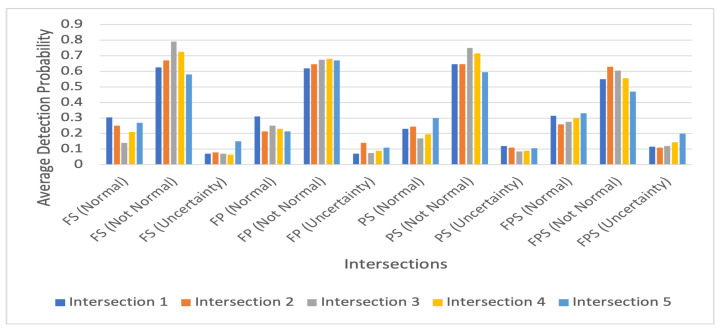
Average detection probability for intruded intersections.

To show how our proposed IDS works to detect both traffic conditions for each intersection, we calculated the average value of the probabilities for both scenarios. Figure 6 and Figure 7 show these average detection results for the normal and intruded traffic conditions, respectively. In Figure 6 and Figure 7, for each intersection, there are 12 bars. The first four bars represent the probability of an intersection being normal, bars five to eight denote the probability of an intersection being not normal, and the last four bars show the uncertainty values. For the normal traffic conditions, as with the results shown in Table 1 and Table 3, for FS, the detection probabilities shown in Figure 6 are higher and above or equal to 0.62 for all interactions except Intersection 3. For these intersections for FSP, Figure 6 also shows higher uncertainty values. However, for the intruded traffic conditions, the probability values for FSP for all intersections are less than those of the others having higher uncertainty values except for Intersection 4.

#### 4.3.2. Class Separability

As we know, the wider separation gap between the value of two decisive parameters makes the system robust and decisions more accurate. For this reason, to show the robustness of our proposed IDS in deciding whether an intersection is normal or not normal, we calculated the separation gap between the average probability values for all pieces of evidence being normal or not normal for any particular intersection.

**Figure 8:**
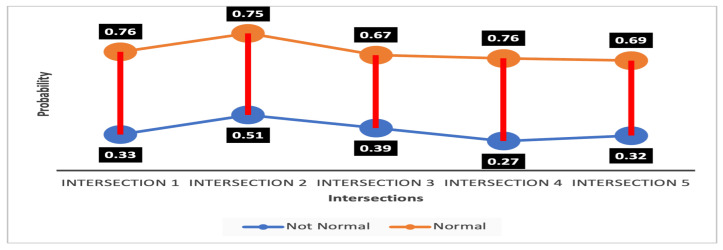
Separation gap between the probability of intersection being normal.

**Figure 9:**
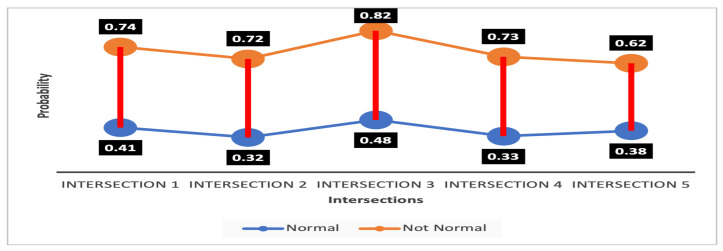
Separation gap between the probability of intersection being intruded.

The separation band of the values of an intersection being normal or intruded is shown in Figure 8 and Figure 9, respectively. The highest gap calculated is for Intersection 4, shown in Figure 8, which has a probability of being not normal of 0.76, and of being normal of 0.27. So, the gap is 0.59. The lowest gap is for Intersection 5, shown in Figure 9. The probability of being normal is 0.615, and not normal is 0.38, and thus the lowest gap is 0.235. The wide separation gap ranging from 0.235 to 0.59 vindicates that our system can conveniently differentiate the normal and intruded intersections.

#### 4.3.3. Detection Performance

We demonstrated the performance of the proposed model in terms of its ability to detect intruded and non-intruded scenarios accurately. For this, we calculated the value of four performance metrics—sensitivity, specificity, accuracy, and F1-Score —using Equations (13) and (14); this is shown in Table 5.

Table 5 displays the performance metrics of different models based on the combination of input features (flow rate, phase time, and vehicle speed). The performance of each model was evaluated using True Positive, True Negative, False Positive, False Negative, sensitivity, specificity, accuracy, and F1-Score. Five different instances (1 to 5) are reported for each model, possibly indicating different test sets or cross-validation results.

1. Flow rate and phase time:
  The model utilising flow rate and phase time as features has sensitivity values ranging from 0.79 to 0.86, specificity values between 0.61 and 0.83, accuracy values varying from 0.77 to 0.85, and F1-Scores ranging from 0.70 to 0.84. This model seems to have a relatively good overall performance with varying levels of specificity.
2. Phase time and vehicle speed:
  This model, based on phase time and vehicle speed, shows sensitivity values between 0.81 and 0.88, specificity values ranging from 0.72 to 0.78, accuracy values varying from 0.79 to 0.85, and F1-Scores ranging from 0.76 to 0.82. The performance of this model is consistent and generally good across all instances.
3. Flow rate and vehicle speed:
  For the model using flow rate and vehicle speed as features, sensitivity values range from 0.81 to 0.87, specificity values are between 0.72 and 0.77, accuracy values vary from 0.79 to 0.83, and F1-Scores range from 0.77 to 0.81. This model demonstrates a fairly consistent and good performance across all instances, with a slightly lower specificity compared to the phase time and vehicle speed model.
4. Flow rate, phase time, and vehicle speed:
  The model that includes all three features—flow rate, phase time, and vehicle speed—has sensitivity values ranging from 0.78 to 0.83, specificity values between 0.52 and 0.62, accuracy values varying from 0.73 to 0.78, and F1-Scores ranging from 0.63 to 0.71. This model shows a drop in performance, especially in specificity, when compared to the other models.
  The models with the best performance are those that utilise two features: phase time and vehicle speed or flow rate and vehicle speed. The phase time and vehicle speed model shows a slightly better performance than the flow rate and vehicle speed model, with higher specificity values. The model using all three features (flow rate, phase time, and vehicle speed) exhibits the lowest performance, especially in specificity. Based on these findings, it is recommended to use the phase time and vehicle speed model or the flow rate and vehicle speed model for further analysis or deployment.

Our system is able to detect most of the normal and intruded traffic conditions used in our simulation successfully. Due to the dynamic characteristics, the traffic condition deviates highly from the ideal condition. These dynamic characteristics cause some False Positives and False Negatives. The results presented in Table 5 show that none of the pieces of evidence achieve a superior performance in terms of all metrics. This is because, as mentioned before, there are some situations in which our system could not detect normal traffic conditions accurately.

## 5. Conclusions

In this paper, we introduced a technique to detect intrusion at the traffic signal of an ITS. Our technique is based on the real-time observation of traffic parameters, namely, traffic flow, average vehicle speed, phase time of the signal, and their historical values. Using the past and currently-observed values, probabilistic estimation of those parameters being normal or abnormal was derived using the DS theory. We also used an approach based on Shannon’s entropy to incorporate the uncertainty of observed data in the detection system. To validate the efficacy of the proposed IDS, simulation scenarios were built using the SUMO platform and the real road network of Melbourne CBD, utilising the actual data from road transport authority on traffic flow, phase time, and vehicle speed. Intrusion scenarios were created by intentional perturbation of those traffic parameters and the proposed system’s capability to identify traffic signal intrusion was assessed in terms of the widely used metrics of detection accuracy, sensitivity, specificity, and F1-Score. The proposed IDS demonstrated an overall accuracy of 79.3% for intruded traffic conditions. Further investigation into the behaviour of the system showed that detection fails if the intrusion is very short-lived in the range of one to two cycles only. This is because such short intrusion fails to produce enough impact in the traffic system to cause a significant deviation from normal data.

The next-generation ITS will be highly complex, comprising autonomous and semi-autonomous vehicles, intelligent sensors connected wirelessly to TMS infrastructure, and all requiring ultra-low latency interactions among themselves and with road-side infrastructure. All these expose ITS to new attacks and vulnerabilities. Future work will focus on improving the detection of intrusion and types of intrusion.

## Figures and Tables

**Table 1:** Detection results for Scenario 1. In the simulation, all intersections operated in normal conditions. The values that are highlighted in bold represent the combination that produces the best outcome.

	Normal (*N*)				Not Normal (¬ *N*)				Uncertainty (*N* ∨¬ *N*)			
	**FS**	**FP**	**PS**	**FPS**	**FS**	**FP**	**PS**	**FPS**	**FS**	**FP**	**PS**	**FPS**
1	0.58	0.65	0.71	**0.76**	0.3	0.29	0.27	**0.16**	0.12	0.06	**0.02**	0.08
2	**0.75**	0.58	0.64	0.72	0.23	0.31	0.29	**0.17**	**0.02**	0.11	0.07	0.11
3	0.48	0.52	**0.67**	0.58	0.39	0.36	0.29	**0.21**	0.13	0.12	**0.04**	0.21
4	0.61	0.61	**0.76**	0.63	0.31	0.29	**0.21**	0.25	0.08	0.1	**0.03**	0.12
5	0.57	0.52	0.58	**0.59**	0.32	0.39	0.3	**0.22**	0.11	**0.09**	0.12	0.19

**Table 2:** Detection results for Scenario 2. In the simulation, all intersections were intuitively intruded (not normal). The values that are highlighted in bold represent the combination that produces the best outcome.

	Normal (*N*)				Not Normal (¬ *N*)				Uncertainty (*N* ∨¬ *N*)			
	**FS**	**FP**	**PS**	**FPS**	**FS**	**FP**	**PS**	**FPS**	**FS**	**FP**	**PS**	**FPS**
1	0.41	0.38	**0.24**	0.41	0.51	0.53	**0.64**	0.5	**0.08**	0.09	0.12	0.09
2	0.32	**0.27**	0.32	0.38	0.62	**0.64**	0.57	0.56	**0.06**	0.09	0.11	**0.06**
3	**0.27**	0.48	0.32	0.46	**0.66**	0.44	0.62	0.43	0.07	0.08	**0.06**	0.11
4	**0.19**	0.24	0.21	0.33	**0.72**	0.69	0.71	0.55	0.09	**0.07**	0.08	0.12
5	0.23	**0.17**	0.26	0.28	0.63	**0.72**	0.62	0.54	0.14	**0.11**	0.12	0.18

**Table 3:** Detection results for Scenario 3. In the simulation, all intersections operated in normal conditions. The values that are highlighted in bold represent the combination that produces the best outcome.

	Normal (*N*)				Not Normal (¬ *N*)				Uncertainty (*N* ∨¬ *N*)			
	**FS**	**FP**	**PS**	**FPS**	**FS**	**FP**	**PS**	**FPS**	**FS**	**FP**	**PS**	**FPS**
1	0.68	0.61	0.59	**0.74**	0.27	0.33	0.35	**0.2**	0.05	0.06	0.04	0.06
2	0.42	0.48	0.50	**0.51**	**0.51**	0.42	0.51	**0.32**	0.07	0.1	**0.06**	0.12
3	0.65	0.58	**0.66**	0.51	**0.30**	0.36	0.34	0.47	0.05	0.06	0.06	**0.03**
4	0.67	0.71	0.65	**0.72**	0.26	0.20	0.26	**0.16**	**0.04**	0.09	0.09	0.13
5	0.65	0.63	0.67	**0.69**	0.33	0.35	0.30	**0.25**	**0.02**	**0.02**	0.03	0.06

**Table 4:** Detection results for Scenario 4. In the simulation, all intersections were intuitively intruded (not normal). The values that are highlighted in bold represent the combination that produces the best outcome.

	Normal (*N*)				Not Normal (¬ *N*)				Uncertainty (*N* ∨¬ *N*)			
	**FS**	**FP**	**PS**	**FPS**	**FS**	**FP**	**PS**	**FPS**	**FS**	**FP**	**PS**	**FPS**
1	**0.20**	0.24	0.22	0.22	**0.74**	0.71	0.65	0.6	0.06	**0.05**	0.12	0.14
2	0.18	0.16	0.17	**0.14**	0.71	0.65	**0.72**	0.70	**0.10**	0.19	0.11	0.16
3	**0.01**	0.02	0.02	0.09	**0.92**	0.91	0.88	0.78	0.08	**0.07**	0.11	0.13
4	0.23	0.22	**0.18**	0.27	**0.73**	0.67	0.72	0.56	**0.04**	0.11	0.10	0.17
5	0.31	**0.26**	0.34	0.38	0.53	**0.62**	0.57	0.40	0.16	0.11	**0.09**	0.22

**Table 5:** Detection results in terms of standard performance metrics. Here, TP = (True Positive), TN = (True Negative), FP = False Positive, FN = False Negative, Sen. = Sensitivity, Spec. = Specificity, Acc. = Accuracy, and F1 = F1-Score.

		TP	TN	FP	FN	Sen.	Spec.	Acc.	F1
FP	1	1960	678	122	536	0.79	0.85	0.80	0.82
	2	2052	306	194	444	0.82	0.61	0.79	0.70
	3	2316	664	136	380	0.86	0.83	0.85	0.84
	4	2157	513	287	439	0.83	0.64	0.79	0.72
	5	2324	505	295	572	0.80	0.63	0.77	0.71
PS	1	2352	619	181	414	0.85	0.77	0.83	0.81
	2	2025	578	222	471	0.81	0.72	0.79	0.76
	3	2435	626	174	382	0.86	0.78	0.85	0.82
	4	2187	606	194	309	0.88	0.76	0.85	0.81
	5	2343	582	218	453	0.84	0.73	0.81	0.78
FS	1	2074	577	223	322	0.87	0.72	0.83	0.79
	2	2295	612	188	401	0.85	0.77	0.83	0.81
	3	2377	613	187	512	0.82	0.77	0.81	0.79
	4	1866	589	211	430	0.81	0.74	0.79	0.77
	5	2203	613	187	393	0.85	0.77	0.83	0.81
FPS	1	2126	458	342	612	0.78	0.57	0.73	0.66
	2	2052	473	327	444	0.82	0.59	0.77	0.69
	3	2352	466	334	472	0.83	0.58	0.78	0.69
	4	1826	413	387	417	0.81	0.52	0.74	0.63
	5	2192	496	304	452	0.83	0.62	0.78	0.71
Overall						0.82	0.71	0.79	0.80

## Data Availability

Publicly available datasets were analyzed in this study. This data can be found here: [https://vicroadsopendata-vicroadsmaps.opendata.arcgis.com/].
